# Gene expression signatures of morphologically normal breast tissue identify basal-like tumors

**DOI:** 10.1186/bcr1608

**Published:** 2006-10-20

**Authors:** Greg Finak, Svetlana Sadekova, Francois Pepin, Michael Hallett, Sarkis Meterissian, Fawaz Halwani, Karim Khetani, Margarita Souleimanova, Brent Zabolotny, Atilla Omeroglu, Morag Park

**Affiliations:** 1 Molecular Oncology Group, McGill University Health Centre, 687 Pine Ave, West, H3A 1A1, Quebec, Canada; 2 Department of Biochemistry, McGill University, 3655 Promenade Sir William Osler, H3G 1Y6, Montreal, Quebec, Canada; 3 McGill Centre for Bioinformatics, McGill University, 3775 University Street, H3A 2B4, Montreal, Quebec, Canada; 4 Breast Cancer Functional Genomics Group, McGill University, 3775 University Street, H3A 2B4, Montreal, Quebec, Canada; 5 School of Computer Science, McGill University, 3480 University Street, H3A 2A7, Montreal, Quebec, Canada; 6 Department of Surgery, McGill University, Montreal, 687 Pine Avenue West, H3A 1A1, Quebec, Canada; 7 School of Medicine, McGill University, Montreal, 687 Pine Avenue West, H3A 1A1, Quebec, Canada; 8 Department of Anatomical Pathology, Sunnybrook Health Sciences Center, 2075 Bayview Avenue, M4N 3M5, Ontario, Canada; 9 School of Pathology, McGill University, 3775 University Street, H3A 2B4, Montreal, Quebec, Canada; 10 Department of Surgery, Grace General Hospital, 300 Booth Drive, R3J 3M7, Winnipeg, Manitoba, Canada; 11 Department of Oncology, McGill University, 546 Pine Ave. W, H2W 1S6, Montreal, Quebec, Canada

## Abstract

**Introduction:**

The role of the cellular microenvironment in breast tumorigenesis has become an important research area. However, little is known about gene expression in histologically normal tissue adjacent to breast tumor, if this is influenced by the tumor, and how this compares with non-tumor-bearing breast tissue.

**Methods:**

To address this, we have generated gene expression profiles of morphologically normal epithelial and stromal tissue, isolated using laser capture microdissection, from patients with breast cancer or undergoing breast reduction mammoplasty (*n *= 44).

**Results:**

Based on this data, we determined that morphologically normal epithelium and stroma exhibited distinct expression profiles, but molecular signatures that distinguished breast reduction tissue from tumor-adjacent normal tissue were absent. Stroma isolated from morphologically normal ducts adjacent to tumor tissue contained two distinct expression profiles that correlated with stromal cellularity, and shared similarities with soft tissue tumors with favorable outcome. Adjacent normal epithelium and stroma from breast cancer patients showed no significant association between expression profiles and standard clinical characteristics, but did cluster ER/PR/HER2-negative breast cancers with basal-like subtype expression profiles with poor prognosis.

**Conclusion:**

Our data reveal that morphologically normal tissue adjacent to breast carcinomas has not undergone significant gene expression changes when compared to breast reduction tissue, and provide an important gene expression dataset for comparative studies of tumor expression profiles.

## Introduction

Despite significant advances in breast cancer treatment, 26% of patients with early disease develop metastasis and succumb to the disease [1]. None of the current prognostic indicators can reliably predict the outcome for such patients [2-6]. Microarrays have been widely used for expression profiling of breast cancer and other malignancies and, because of their genome-wide nature, they allow for the identification of gene expression changes that have occurred between normal and tumor breast tissues. Using these approaches, several studies have successfully identified breast cancer subtypes and prognostic markers; however, the utility of such markers in the clinic remains open [7-11].

The majority of studies focusing on breast have used heterogeneous material from whole tissue sections with a few exceptions where epithelial cells have been specifically isolated [12]. The presence of loss of heterozygosity in normal stromal breast tissue adjacent to, and distant from, the tumor site has been demonstrated, suggesting that changes in stroma may have occurred [13]. Since surgery is the standard of care, normal cells harboring alterations that may be relevant to cancer progression may remain and, thus, could have important clinical implications.

The normal human breast consists of ductal epithelium and surrounding stroma. The stroma consists of two compartments (intralobular stroma and extralobular stroma), accounts for more than 80% of the breast volume, and provides nutrition and structural support for the normal epithelium. Carcinoma of the breast, as well as benign hyperplastic conditions, are thought to originate from epithelial cells or progenitor epithelial cells of the terminal duct-lobular unit [14]. However, growing evidence indicates that stroma may play an important role in cancer initiation and progression [15-17]. Little is known regarding gene expression profiles in morphologically normal breast stroma or epithelium adjacent to breast tumor tissue.

At the clinical level, normal tissue is defined as morphologically normal. Laser capture microdissection (LCM) allows one to isolate nearly pure cell populations from a heterogeneous environment, and the material is suitable for microarray gene expression analysis [12,18,19]. This approach has allowed the comparison of gene expression profiles between normal human breast epithelium and tumor tissue [12]. Epithelium derived from regions of the breast adjacent to tumor, considered normal by all histological and clinical standards, has been shown to have a distinct gene expression profile from tumor tissue [12]. However, in these cases sample sizes have been small when comparing reduction and adjacent tissue (*n *= 3 reduction samples) and, furthermore, stroma was not considered [12]. Thus, knowledge of gene expression patterns in normal tissue would be invaluable to improve the precision of gene expression signatures for poor or good prognosis.

In the present study, LCM was used to dissect normal epithelium and normal stroma derived from patients undergoing breast reduction mammoplasty or surgical treatment of breast cancer. Gene expression profiles reveal that morphologically normal stroma and epithelium from breast cancer patients are not statistically distinct from epithelium and stroma isolated from reduction mammoplasties and do not possess gene expression changes associated with standard clinical characteristics.

## Materials and methods

### Clinical data

Clinical data were collected for the samples from the Breast Cancer Functional Genomics Group clinical database. Cellular and fibrotic stroma were identified by visual inspection of hematoxylin and eosin stained tissue sections under a microscope. Cellular stroma was defined as tissue with more than 1,000 stroma cells uniformly distributed throughout the field of view (4× magnification), while fibrotic stroma was defined as tissue with less than 800 stroma cells in the field of view (4× magnification) and concentrated primarily around the ducts.

### Tissue collection and staining procedures

All tissue specimens and associated clinical data were collected at McGill University Health Center (Montreal, Canada) between 2000 and 2004 in accordance with the protocols approved by the research ethics committee. Patient consent was obtained on an individual basis for all patients participating in this study. Of 44 patients selected for the study, 34 patients had invasive ductal carcinoma and 10 were healthy donors undergoing reduction mammoplasty. Tissue samples were collected within 30 minutes after surgery, embedded in TissueTek OCT (Somagen, Edmonton, Alberta, Canada) and stored in liquid nitrogen until use. Frozen specimens were cryosectioned in 10-micron slices, stained using a hematoxylin and eosin staining protocol and dehydrated in ethanol and xylene as recommended by the LCM manufacturer (Arcturus, Mountain View, CA, USA). Following dehydration, the slides were air dried for 20 minutes and subjected to LCM. All normal tissues adjacent to tumor were microdissected from regions at least 2 mm away from tumor margins. Normal and adjacent stroma were sampled exclusively from the extralobular stromal compartment.

### LCM, RNA extraction and linear amplification

All tissues included in this study were re-examined by a clinical pathologist dedicated to the project. Tissue specimens were microdissected into epithelium and stroma using a PixCell IIe LCM system (Arcturus). All microdissections were performed within three hours following tissue staining. Total RNA was extracted from each population of microdissected cells using a GITC (guanidinium isothiocyanate) extraction protocol. Briefly, LCM caps were incubated for 5 minutes (room temperature) in 200 μl GITC extraction buffer (4 M GITC, 25 mM sodium citrate pH 7.0, 0.1 M β-mercaptoethanol, 0.5% N-lauroylsarcosine) supplemented with 1.6 μl β-mercaptoethanol. Subsequently, 20 μl of 2 M NaOAc, pH 4.0, 220 μl of water-saturated phenol and 60 μl of chloroform-isoamyl alcohol (23:1) were added to the extraction buffer. Following 15 minutes incubation on ice and centrifugation (12,000 rpm, 15 minutes) the aqueous phase was removed and RNA was precipitated with 2 μl glycogen (GenHunter, Nashville, Tennessee, USA) and 200 μl isopropanol. Samples were placed at -80°C for 30 minutes and centrifuged at 4°C (12,000 rpm) for 30 minutes to pellet RNA. Pellets were washed with 70% ethanol, air dried and subjected to DNAseI treatment (Roche, Basel, Switzerland). DNAseI treatment was performed in the presence of an RNase inhibitor (Invitrogen, Carlsbad, California, USA). Subsequently, samples were re-extracted as described above and re-suspended in 10 μl of diethylpyrocarbonate-treated water. RNA was quantified using a RiboGreen assay (Molecular Probes, Carlsbad, California, USA). Subsequently, 2 to 4 ng of total RNA was subjected to two rounds of T7 linear amplification using Ambion Amino Allyl MessageAmp kit (Ambion, Austin, Texas, USA) and labeled with Cy3 and Cy5 dyes according to the manufacturer's procedure. Prior to microarray hybridizations, amplified products were quantified using a spectrophotometer (Nanodrop, Wilmington, Delaware, USA) and subjected to BioAnalyzer to assay for quality (Agilent Technologies, Santa Clara, California, USA).

### Microarray hybridization

Whole Human Genome 44 K arrays (Agilent Technologies, product G4112A) were used for all experiments. RNA samples (500 ng) were subjected to fragmentation followed by 18 h hybridization, washing, and scanning (Agilent Technologies, model G2505B) according to the manufacturer's protocol (manual ID #G4140-90030). Samples were hybridized against Universal Human Reference RNA (Stratagene, ID #740000, La Jolla, California, USA). Duplicate hybridizations were performed for all samples using reverse-dye labeling.

### Immunohistochemistry

Candidate tissue markers were validated by immunohistochemistry. Frozen tissue sections (10 μm thick) were defrosted at room temperature for 30 s, fixed in acetone (room temperature, 10 minutes) and air dried for 2 minutes. Subsequently, tissue sections were blocked with Peroxidase Blocking Reagent (DakoCytomation, Glostrup, Denmark). Primary antibodies were diluted at 1:50 and 1:15 for anti-c-kit (polyclonal rabbit anti-human CD117, DakoCytomation), and anti-CD31 (polyclonal mouse anti-human, DakoCytomation) and applied to the tissue sections for 45 and 15 minutes, respectively. Following a brief wash with TBS-T (tris-buffered saline tween-20), secondary antibodies were applied for 30 and 20 minutes, respectively. Labeled polymer-HRP anti-rabbit (EnVision+ System HRP(DAB), DakoCytomation) was used as a secondary antibody for c-kit staining and labeled polymer-HRP anti-mouse (EnVision+ System HRP(DAB), DakoCytomation) for CD31 staining. After a short wash with TBS-T, DAB Substrat-Chromogen Solution (EnVision+^® ^System HRP(DAB) DakoCytomation) was applied for up to 5 minutes for color development.

### Data preprocessing, normalization, and quality control

Microarray data were feature extracted using Feature Extraction Software (v. 7.11) from Agilent with the default parameters. Raw data were uploaded to the NCBI Gene Expression Omnibus database (GEO) and is accessible as data series GSE4823. Outlier features on arrays were flagged by the software. Arrays were required to have an average raw signal intensity of 1,000 in each channel, and a signal to noise ratio above 16 per channel. MvA plots were examined for signs of hybridization or labeling problems. Replicate arrays were required to have a concordance above 0.944. This level was established empirically using sets of known good replicate arrays in our database.

Data preprocessing and normalization were automated using the BIAS system [20]. Raw feature intensities were background corrected using the RMA background correction algorithm [21,22]. Resulting expression estimates were converted to log2-ratios. Within array normalization was performed using spatial and intensity-dependent loess [23]. Median absolute deviation scale normalization was used to normalize between arrays [24].

### Class discovery

Using class discovery under correlation distance and Euclidean distance metrics, 10,000 bootstrap iterations were performed to assess the significance of the observed clusters using the pvclust package for R[25]. Multidimensional scaling was applied to reduce the dimensionality of the data and permit visualization. Chi-square tests and logistic regression were applied to discrete and continuous variables, repsectively, to test for association with data partitions (clusters). The variables tested included estrogen receptor (ER) status, progesterone receptor (PR) status, lymph node (LN) status, HER2 receptor status, menopause status, age, grade, tumor size, and recurrence.

### Class distinction

Both the linear models for microarray analysis (LIMMA) and significance analysis of microarrays (SAM) algorithms were used to identify differentially expressed gene sets from which to build class predictors [26-29]. Genes from LIMMA were filtered for significance, (false discovery rate adjusted p value ≤ 0.01), fold change (≥2.0), intensity above background (A > 6.0), while genes identified by SAM were filtered by significance (q ≤ 0.3), fold change (≥2.0), and intensity (A > 6.0).

### Class prediction

The prediction around medoids (PAM) algorithm was used to build predictors based on the filtered gene sets [30]. Cross validation was used to test the predictors. This procedure included independent selection of candidate gene sets for each cross validation step. Differentially expressed genes were mapped onto Gene Ontology (GO), and GO terms were tested for overrepresentation using the hypergeometric distribution [26].

### Assessing patient specific gene expression effects

We wanted to assess the relative contribution of different factors to the overall variability of gene expression observed in our data. Principal component analysis allows one to succinctly summarize data in a reduced number of dimensions (principal components) [31]. The principal components are ordered by the amount of variation (or signal) in the data that they explain. We performed principal component analysis on the patient matched adjacent stroma and epithelial data. Consecutive sequences of the first 10 principal components were tested for association with clinical characteristics using multivariate analysis of variance (MANOVA). Bonferroni multiple testing correction was applied to the resulting p values [31].

### Identification of tissue markers

LIMMA was used to identify differentially expressed genes between tissues in individual patients and obtain expression estimates for the matched data ([28,32]. Genes not exhibiting differential expression in at least 50% of samples were excluded from further analysis (B-statistic > 0). A paired t-test was used to identify genes whose patient-matched LIMMA expression estimates were significantly different from zero over the panel of patients (false discovery rate adjusted p value < 1e-5).

### Comparison with publicly available cancer datasets

The expression of gene signatures from a number of publicly available datasets was examined in normal tissue.

The stroma-specific and epithelium-specific gene lists identified by Allinen and colleagues [33] contained 231 and 97 unique genes, respectively, of which 189 and 89 were located (mapped) successfully on the Agilent chip. The activated and inactivated core serum response (CSR) genes from Chang and colleagues [34] contained 228 and 233 genes, respectively, of which 209 and 211 were mapped to the Agilent array. The intrinsic breast cancer gene list of Sorlie and colleagues [35] contained 553 genes, of which 473 were mapped to the Agilent array. The desmoid type fibromatosis (DTF) and solitary fibrous tumor (SFT) specific gene lists from West and colleagues [36] contained 493 and 293 genes, respectively, of which 415 and 238 were mapped to the Agilent array. Genes that were likely to be expressed in normal breast tissue were selected from these gene sets by selecting genes with variance >1 in the normal tissue data; 7.3% of genes in the normal dataset have variance >1, and enrichment for high variance genes in the various gene sets was measured by a χ^2 ^goodness of fit test.

Genes from the Agilent whole genome arrays were mapped to the Agilent 24 K arrays used in the Netherlands cancer dataset [8]. The 24 K arrays used by Van de Vijver and colleagues [8] contained 24,498 features. Approximately 10,000 contigs on the 24 K array could not be mapped to GenBank identifiers. Of the remaining 14,339 identifiers, 12,112 were mapped to features on the 44 K Agilent array. Expression of the genes from the normal tissue signature was then examined in the 295 breast cancer samples from the Netherlands cancer dataset [8].

### Accession numbers

The GEO accession number of the array data series is GSE4823.

## Results

### Identification of stroma- and epithelium-specific gene expression profiles

**Figure 1:**
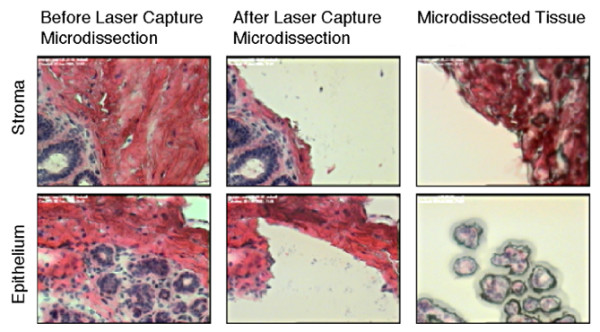
Laser-capture microdissection of epithelium and stroma from normal breast specimens. Frozen tissue sections (10 micron) stained with hematoxylin and eosin.

To determine the gene expression profiles of morphologically normal epithelium and stroma derived from reduction mammoplasties and breast cancer tissue, we integrated the use of LCM and T7-based RNA amplification with DNA microarrays. LCM provides an accurate means by which to isolate morphologically normal epithelium and stroma adjacent to breast cancer that is free from infiltrating tumor cells. This allows gene expression profiles to be generated from specific cell types rather than whole tissue [18]. LCM was used to isolate matched morphologically normal epithelial and stromal cells from 34 patients with invasive ductal carcinoma, and 10 patients who underwent reduction mammoplasty (Figure 1). Patient and tumor characteristics of the selected invasive ductal carcinoma patients are shown in Table 1 (and Additional file 4). In general, 2 to 5 ng of RNA were extracted from dissected normal epithelial ducts and stroma. We, as well as others, have established that T7 linear amplification preserves the ratios of mRNA abundance between mRNA species, provided all samples undergo the same number of amplification rounds [12,37-41].

**Figure 2:**
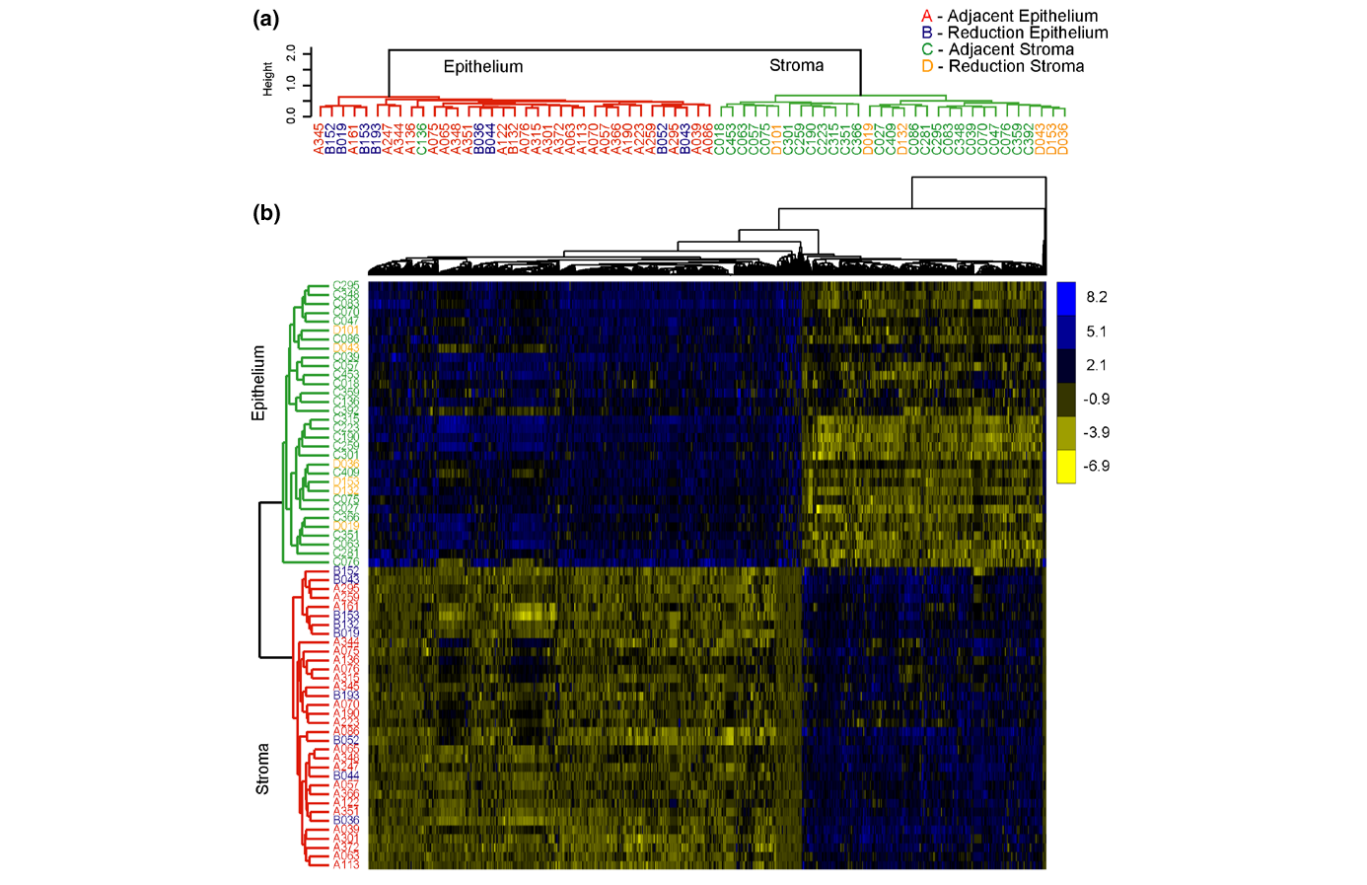
Hierarchical clustering and heatmap showing the segregation of samples by tissue type. **(a) **Hierarchical clustering of normal tissue samples shows segregation by tissue type (red, adjacent epithelium; blue, reduction epithelium; green, adjacent stroma; orange, reduction stroma). **(b) **Heatmap showing tissue specific gene expression clusters.

**Figure 3:**
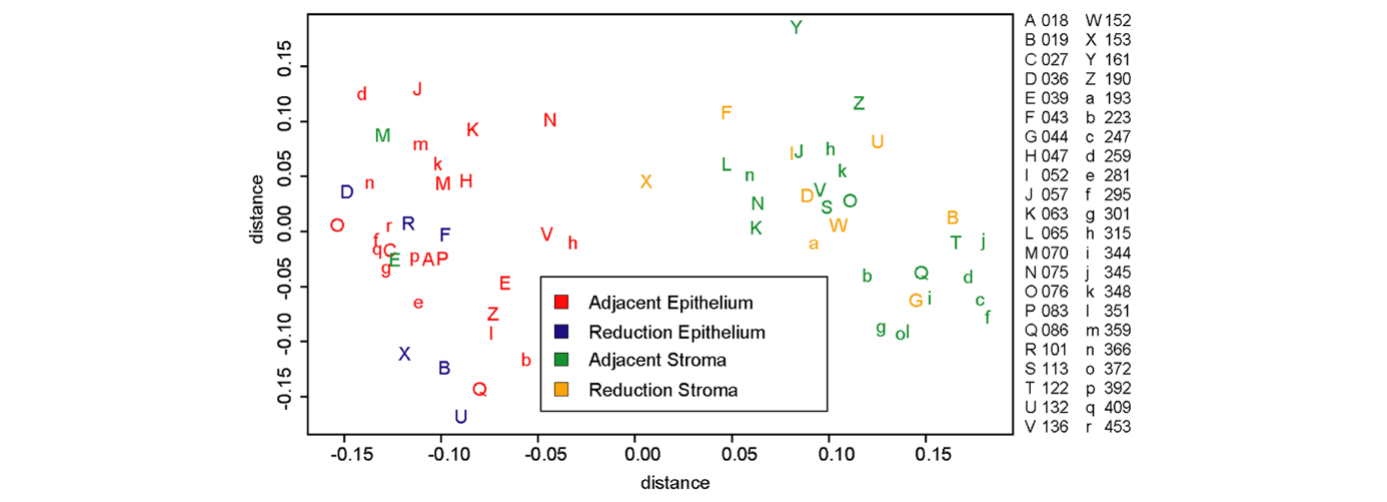
Multidimensional scaling of normal stroma and normal epithelium. Two tissue-specific clusters are observed. Adjacent and reduction tissue do not segregate into separate clusters. The epithelial tissue cluster contains two adjacent stroma sample outliers.

Expression profiling was performed on cells isolated from morphologically normal epithelial and stromal tissue from 34 cases of invasive ductal carcinoma and 10 cases of reduction mammoplasty using Agilent whole genome arrays. A total of 66 samples were analyzed, of which 32 were isolated from stroma (26 from histologically normal ducts adjacent to tumor, and 6 from reduction mammoplasty), and 34 from epithelium (25 from histologically normal ducts adjacent to tumor, and 9 from reduction mammoplasty) (Table 1). Each of the LCM captured samples was interrogated in duplicate on a 44 K genomic feature microarray. Since several studies have suggested that normal stroma as well as morphologically normal terminal duct lobular units from cancer patients undergo loss of heterozygosity [42], we first performed a cluster analysis to determine whether the patient-matched stroma and morphologically normal epithelium were similar to those from reduction mammoplasty patients. After normalization, hierarchical clustering was applied to the 66 samples and the complete panel of genes (44 K genome features). Based on gene expression, the stroma and epithelium clustered according to tissue type (Figure 2a). Stroma surrounding histologically normal ducts from tumor specimens and stroma isolated from reduction mammoplasty clustered together. Similarly, morphologically normal epithelium from tumor specimens co-clustered with epithelium from reduction mammoplasties (Figure 2a). We observed similar tissue-specific clustering when using a multidimensional scaling class discovery approach (Figure 3; see Materials and methods). Only three adjacent stroma samples were found to behave as outliers, clustering with epithelial tissue at the whole genome level, an error rate comparable to other large scale microarray data sets (Figures 2a and 3).

To identify the genes responsible for the tissue-specific clustering observed in Figure 2a, class distinction was applied to identify all genes differentially expressed between tissues. Markers were defined based on patient matched stromal and epithelial samples (22 patients and 44 samples; see Materials and methods; Table 1). In total, 883 markers were identified that showed differential expression between matched epithelium and stroma in at least 50% of individual samples (LIMMA log odds >0), as well as differential expression between pooled epithelium and stroma samples (false discovery rate adjusted p value 1e-5; Additional file 8). Using these markers, hierarchical clustering was applied to the complete sample set (44 patients, 66 samples), and resolved the samples into epithelial and stromal clusters, including correct classification of the three outlier samples (Figure 2b). These genes define a normal tissue gene expression signature.

**Figure 4:**
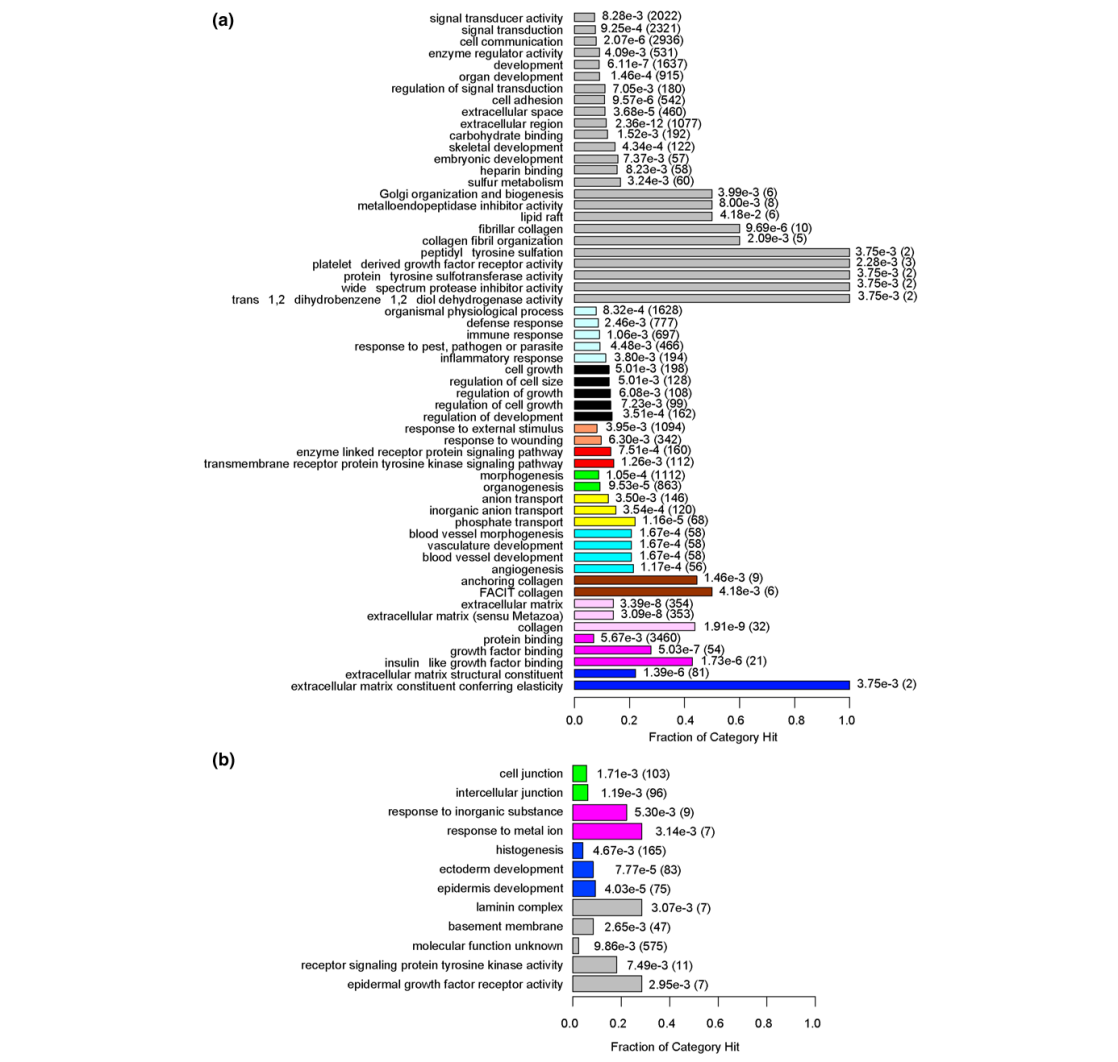
Gene Ontology (GO) categories overrepresented in the normal stroma and normal epithelium gene signatures. **(a) **GO terms overrepresented by genes expressed in normal stroma. **(b) **GO terms overrepresented by genes expressed in normal epithelium. Bars represent the fraction of genes in the category that were expressed. Terms of the same color are related in the GO hierarchy (gray terms are unrelated). P values for significance, and the total number of genes in a category are listed after the bar plot.

**Figure 5:**
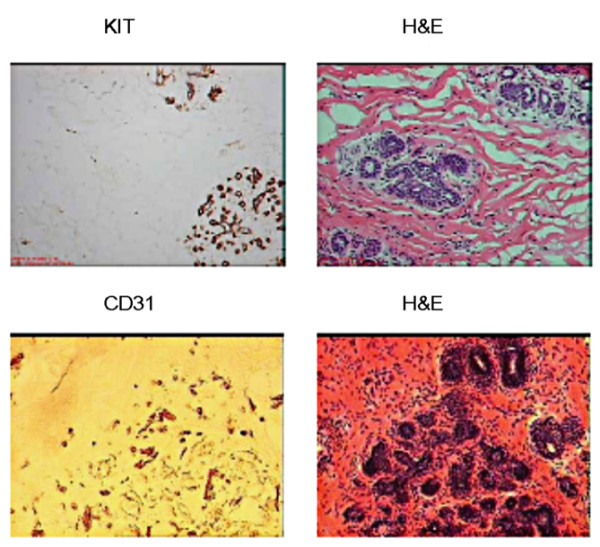
Immunostaining of normal breast tissue with anti-c-kit and anti-CD31. H&E, hematoxylin and eosin.

The complete list of GO terms overrepresented by genes in the normal tissue signature is located in Additional file 9 and summarized in Figure 4. Tissue specific genes in the normal signature include known fibroblast, endothelial, and epithelial genes, as well as potentially novel tissue markers. Epithelium-specific transcripts include genes associated with epithelial cell-cell junctions and the basal lamina, epithelial cell differentiation as well as epidermal growth factor receptor activity (Table 2). Stroma specific genes included extracellular matrix structural constituents, genes with collagen binding activity, and genes involved in angiogenesis and response to wounding (Table 2, Figure 4). Immunohistochemistry for selected proteins using commercially available antibodies demonstrated epithelial-specific expression of Kit, as well as elevated expression of von Willebrand factor and cd31 in stroma, and confirmed the microarray results (Figure 5).

### Normal stroma and epithelial specific gene sets are not predictive of clinical characteristics

**Figure 6:**
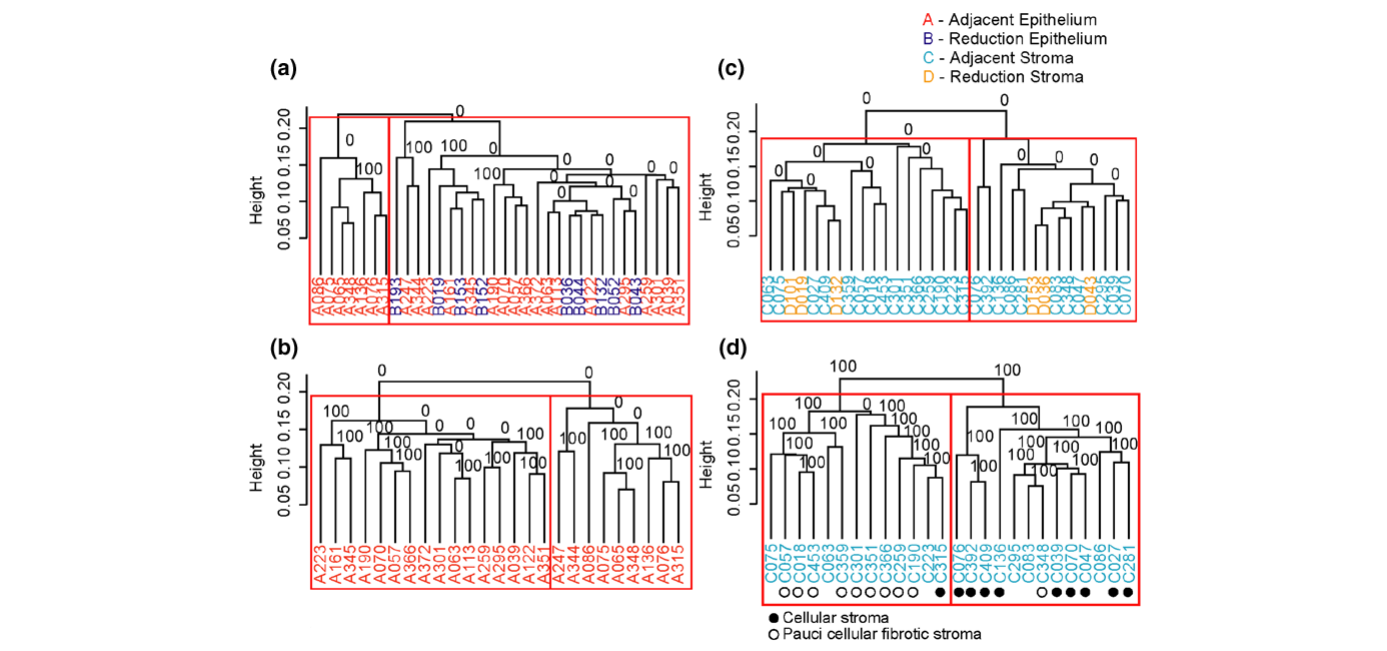
Hierarchical clustering with bootstrapping of adjacent and reduction breast tissues from gene expression data. **(a) **Hierarchical clustering with bootstrapping of adjacent and reduction epithelium. **(b) **Histologically normal adjacent and reduction stroma. **(c) **Histologically normal adjacent epithelium. **(d) **Histologically normal adjacent stroma. We used 10,000 bootstrap iterations to obtain significance scores for the observed clusters. Nodes are labeled with the percentage of times that the cluster is observed by bootstrapping. Only adjacent stroma showed statistically significant clusters at the top level. Red boxes indicate the top-level clusters that were tested for association with clinical characteristics of the samples.

Epithelial and stromal samples were analyzed separately to determine whether there were significant differences within tissue classes between reduction mammoplasty-derived tissue and adjacent morphologically normal tissues isolated from tumor sections. Samples in each tissue class were subjected to hierarchical clustering and subsequent bootstrapping to test for significance using all genes. Although epithelial and stromal samples each show two primary subclusters, these clusters were not statistically significant (Figure 6a,c, respectively). Importantly, adjacent and reduction samples were not associated with the subclusters in either tissue class (p = 0.732 and p = 0.075, respectively, χ^2^ test for association). This analysis was repeated using a subset of genes (filtered by coefficient of variation ≥4), and showed similar results (data not shown).

**Figure 7:**
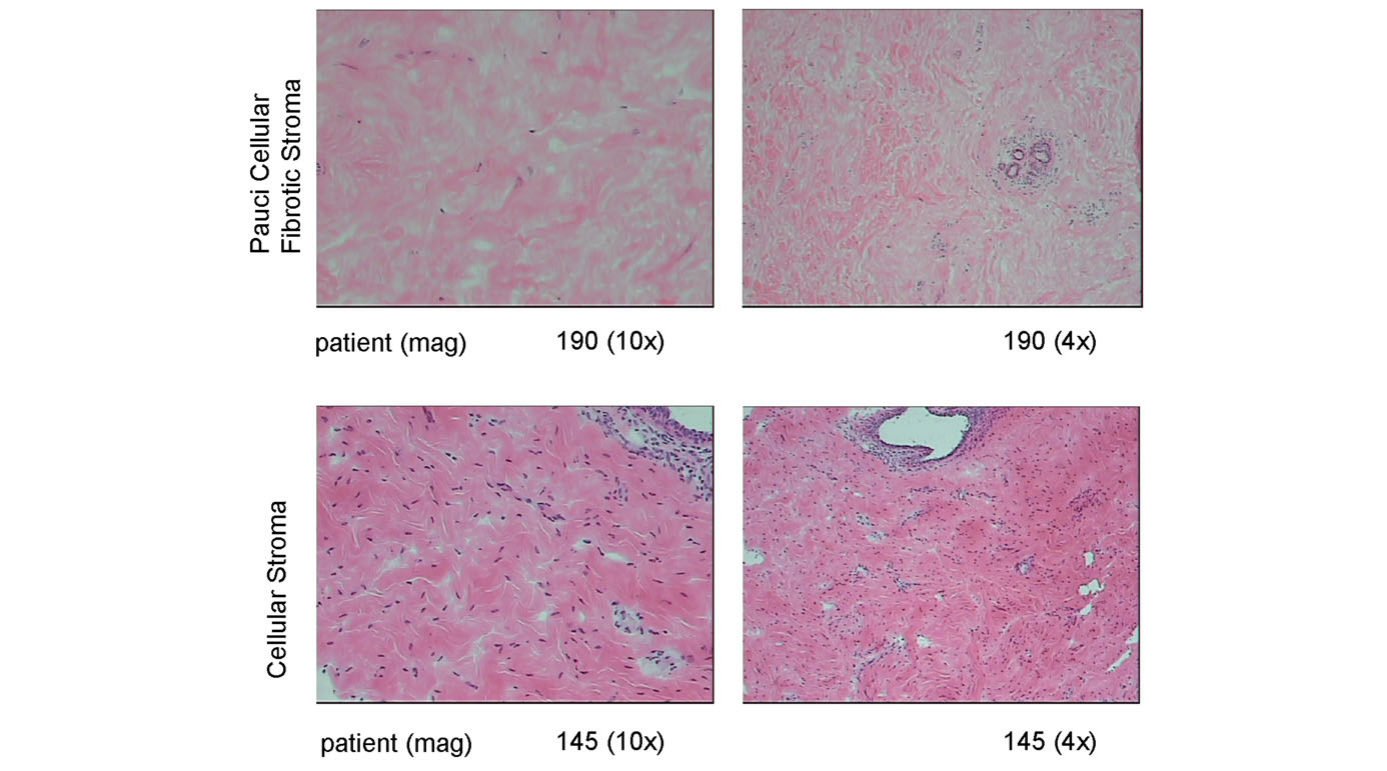
Images of pauci cellular fibrotic and cellular stroma sections from selected patients. Images were taken at 4× and 10× magnification.

Interestingly, morphologically normal adjacent stroma, without reduction mammoplasty samples, was found to consist of two significant subclusters whether using all genes (Figure 6d), or a filtered subset of genes (p = 1.64e-3, Fisher's exact test). These clusters were found to be associated with stromal 'cellularity' (Figure 7, defined in Materials and methods), which was assessed based on the hematoxylin and eosin staining of the normal tissues (p = 5.1e-3 and 2.1e-4, respectively, χ^2^ test for association). A total of 669 genes were identified as differentially expressed between the adjacent stroma clusters using the LIMMA software with a false discovery rate less than 0.01, a fold change of at least 1.9, and a B statistic of at least 30. The majority of these genes were elevated in the pauci cellular fibrotic cluster when compared with the cellular stromal cluster. Furthermore, no association was found between clinical characteristics of the primary tumor, and statistically significant subclusters in either tissue class (p ≤ 0.01, data not shown) (Additional files 10 and 11). In contrast, no significant clusters were found within morphologically normal adjacent epithelium (Figure 6b, Additional file 3).

To determine whether gene expression patterns in normal breast epithelium or stroma derived from breast cancer patients can predict clinical or pathological features of the corresponding cancers, we applied a class prediction [30] approach and constructed tissue specific predictors for ER, PR, HER2, grade, tumor size, age, menopause status, recurrence, and lymph node status (Additional files 2 and 3). We used cross validation at every step of predictor construction [43], including the initial step of candidate gene selection. None of the predictors had low prediction error or low variance, with an average 50% mean prediction error by cross validation (Additional files 2 and 3). This analysis demonstrated that any gene expression differences detected in normal epithlium and stroma were neither associated with, nor predictive of, the clinical characteristics of the primary tumors.

Morphologically normal samples from different individuals are expected to show variations in gene expression due to a number of factors, including noise, differences in tissues, inter-individual variation, potential clinical differences, and the simple fact that different genes are expressed at different levels. Our goal was to identify the relative contribution of each of these sources of variation to our data (Additional file 12, panel A). Principal component analysis and multivariate analysis of variance revealed that the primary sources of variation in the data could be attributed to differences between tissues (Bonferroni corrected p = 7.9e-16, principal components 2 and 3), representing 3.98% of the variation between genes (Additional file 12, panel B), and differences between individuals (Bonferroni corrected p = 4.9e-6, principal components 3 through 8), representing 3.58% of the variation between genes. The majority of the variation in the data (84.58%) could be attributed to variations in expression between genes within a single sample. The strong correlation between arrays introduced by the common reference design of our experiment caused this variation to be common across all arrays (Additional file 13). Together, these effects accounted for 92.13% of the observed variation in the data. The remainder of the variation in gene expression was not associated with any known factors.

### The normal epithelium and stroma expression set identify subtypes of breast carcinoma

The identification of gene expression profiles for morphologically normal stroma and epithelium provide unique datasets that can be used to investigate breast cancer datasets for similarity to the normal tissue profile in order to gain a better understanding of breast cancer expression profiles. When our stroma and epithelium profile was compared to a dataset established by a serial analysis of gene expression (SAGE) approach from dispersed cells from one reduction mammoplasty sample [33], we observed a minimal overlap. Our normal stroma signature (562 unique genes) showed only a 25 gene overlap with that generated by SAGE for a mixture of fibroblast, endothelial and myofibroblast cells (mapped 189 unique genes), and a 2 gene overlap with the epithelium signature (mapped 89 unique genes), whilst our normal epithelium signature (321 unique genes) overlapped by 12 genes with the epithelium signature identified by SAGE. Although the overlaps are statistically significant (p = 1.33e-15 and p = 9.07e-12, respectively, hypergeometric test), the relatively low overlap between the signatures may be due to use of only a single patient in the SAGE data when compared to 44 patients in our dataset and our filtering criteria. However, the fact that no genes are in common between the epithelial gene set and that of the fibroblast data obtained by SAGE supports the purity of both cell populations in these studies.

**Figure 8:**
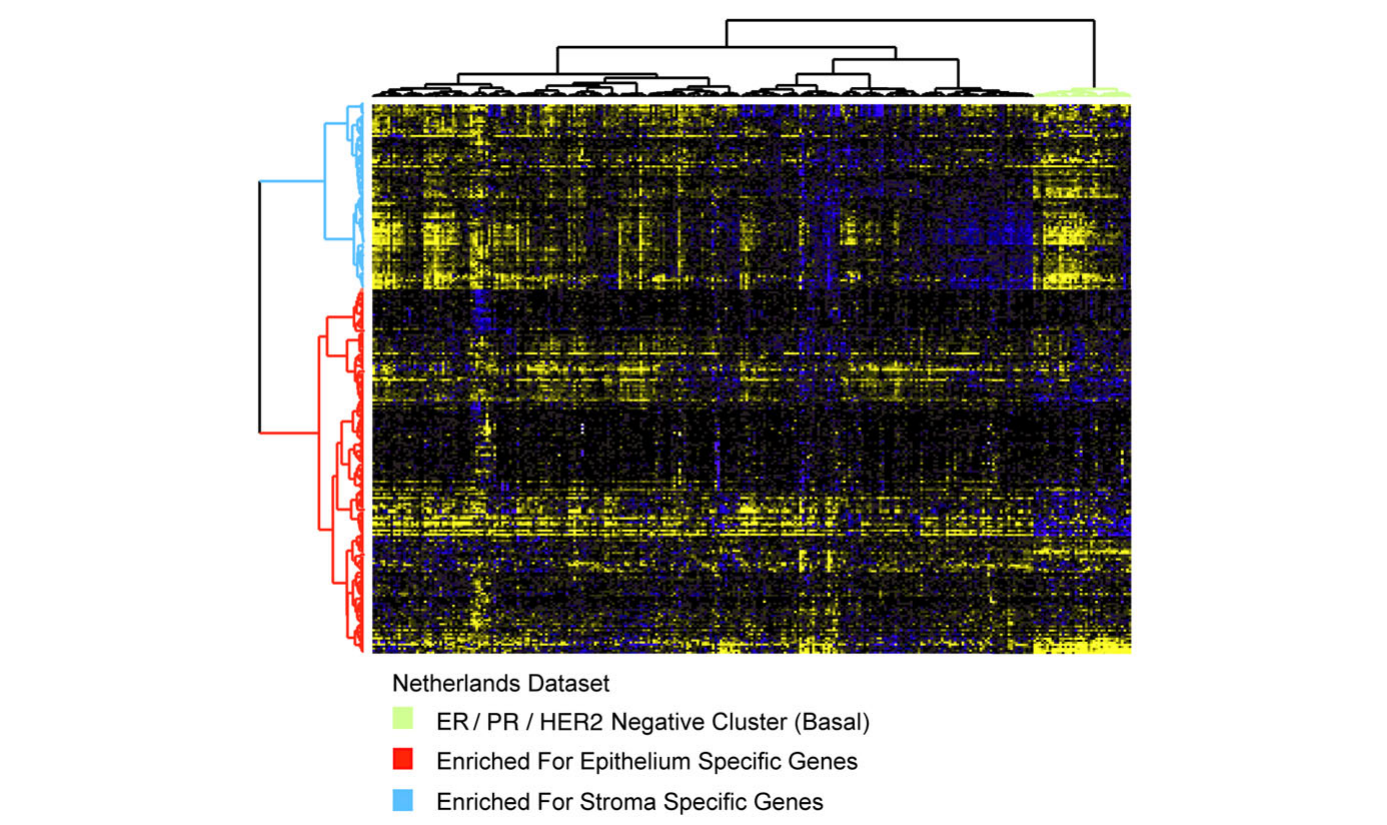
Heatmap of a cancer dataset [8] clustered using the normal gene signature. This signature identifies a distinct cluster of 38 estrogen receptor (ER)/progesterone receptor (PR) negative, HER2 negative samples corresponding to the basal breast cancer subtype [35]. Elevated expression of normal stroma-specific genes appears in a portion of the lumenal and HER2 positive tumors, although this expression does not correlate with the known molecular subtypes of breast cancer.

**Figure 9:**
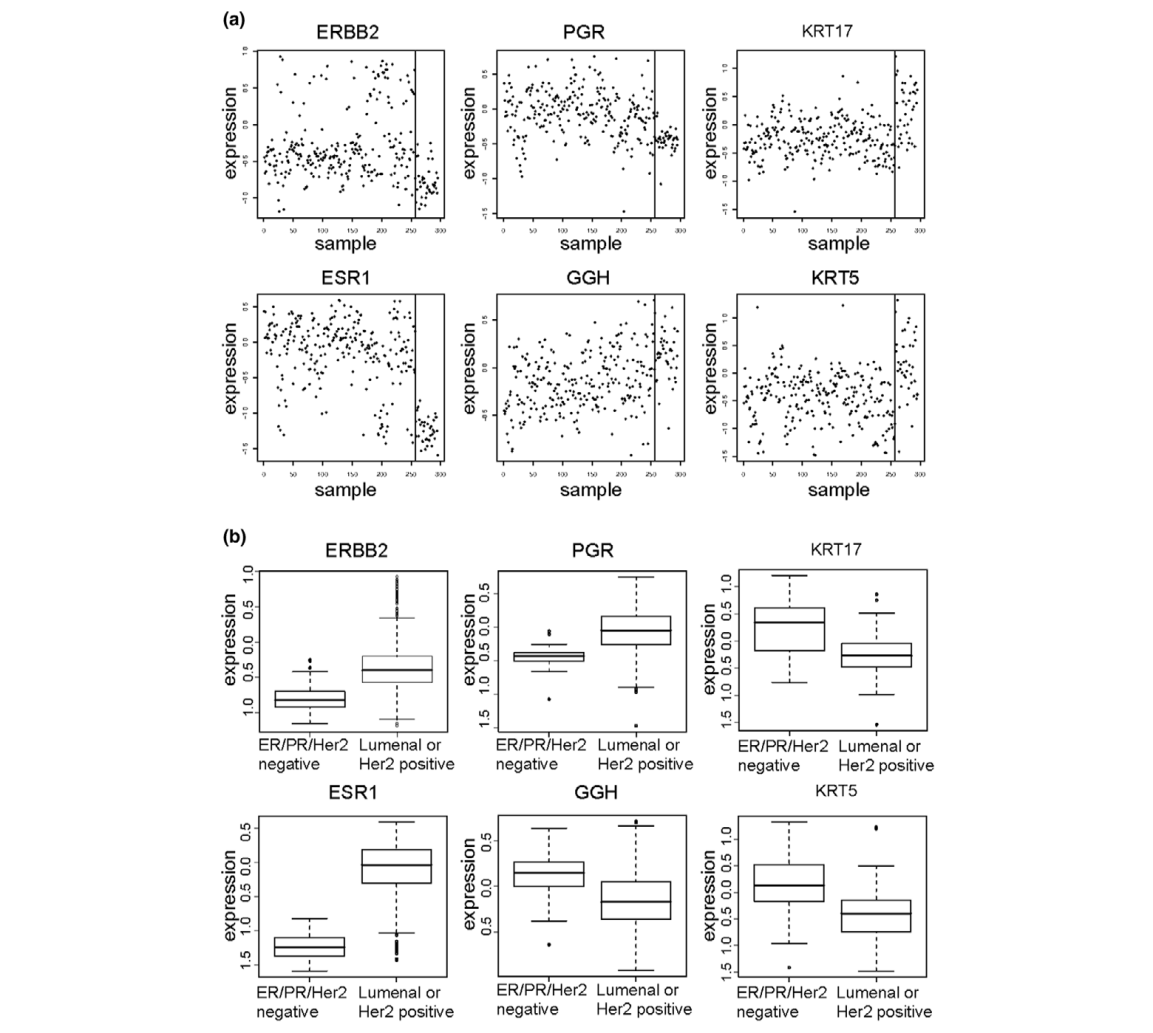
Expression of selected subtype specific markers in the breast cancer dataset [8]. **(a) **The y-axis shows the expression level of the gene, while the x-axis identifies each sample, ordered as in Figure 8. The vertical line shows the separation between sample clusters found in Figure 8. The right-most cluster shows decreased expression of estrogen receptor (ESR), HER2, and progesterone receptor (PGR) in most samples, and increased expression of keratin (KRT) 5 and gamma-glutamyl hydrolase (GGH). These markers are indicative of a mixture of basal-like and normal-like tumor subtypes. **(b) **Box plots showing the distributions of expression for subtype markers in the two observed clusters.

To investigate the implication of the expression profiles generated from normal breast tissue *in situ *to those of tumor related genes in breast cancer, we analyzed the expression of genes in 295 breast carcinomas using a previously published dataset [8]. The normal tissue signature was mapped to 349 genes on the custom 24 K Agilent arrays used for the cancer study [8]. Hierarchical clustering of the 295 patient samples present in the cancer dataset using the genes in our normal tissue (stroma plus epithelium) signature, revealed two primary clusters of samples (Figure 8). Based on tissue specificity defined by our normal signature, the larger cluster showed enrichment for stroma specific genes (p = 0.0038, hypergeometric test) and showed an under-representation of epithelium specific genes (p = 0.001, hypergeometric test). However, this enrichment for stroma specific genes was not ubiquitously observed for all of the 257 tumor samples in the cluster, nor was it found to be associated with either the HER2, luminal A or luminal B tumor subtypes (Figures 8 and 9). In contrast, the smaller cluster showed enrichment for epithelium specific genes (p = 4.16e-13, hypergeometric test) and under-representation of stroma specific genes (p = 4.5e-42, hypergeometric test).

The smaller of the two clusters consisted of 38 samples, which were identified as ER negative, HER2 negative, and PR negative (Figure 9). This ER/HER2/PR negative cluster was found to express many normal and basal subtype specific genes as defined by Sorlie and colleagues [35], including keratin-5, keratin-17, and gamma-glutamyl hydrolase (GGH). Based on expression of these markers, we identified the samples in this cluster as consisting of basal-like and normal-like cancer subtypes as defined previously [35]. The remaining ER negative samples in the cancer dataset were HER2 positive and were located in the larger sample cluster. Notably, the cluster of basal-like and normal-like samples remained when the data was clustered using only our normal epithelium-specific gene set, whereas the cluster was not observed when normal stroma-specific genes were used in clustering (data not shown). This indicated that the basal subtype-specific patient cluster was enriched in genes expressed in normal epithelium when compared with other tumor subtypes.

### Normal stroma is similar to DTF tumors and fibroblasts with an inactivated core serum response

Few datasets have been generated for stroma, and this is the first extensive dataset to be generated from normal stroma. To determine whether our normal stroma data set resembled other gene expression profiles for fibroblasts, a core set of genes shown to be differentially regulated when fibroblasts are stimulated with serum [44] was examined. We identified genes from the CSR profiles that were expressed in normal tissue (Additional files 6 (panel D) and 7) using a variance filtering criteria (see Materials and methods). Of the unstimulated fibroblast genes expressed in normal tissues, 84% were expressed in stroma, while 16% were expressed in epithelium, while the majority of genes activated in wounding were not expressed in either tissue (Additional file 6, panel C). These results indicate that both normal adjacent stroma and normal reduction stroma have expression profiles more similar to unstimulated fibroblasts.

To investigate the similarity of our normal stromal profile to that of fibroblastic tumors, normal stroma and epithelium expression profiles were compared to the gene signatures of DTF and SFTs [36]. Normal stroma samples expressed significantly more DTF-specific genes than expected by chance (p ≤ 2e-16, χ^2 ^goodness of fit test), while the number of SFT-specific genes was marginally significant (p = 0.038, χ^2 ^goodness of fit test) (Additional files 6 (panel A), and 7). Interestingly, normal stroma showed a statistically significant enrichment for expression of DTF-specific genes (p = 2.48e-5) (Additional file 6, panel B).

## Discussion

Knowledge of the normal breast microenvironment in which a cancer develops is important in understanding cancer biology. However, gene expression patterns of normal stroma and epithelium in human breast cancers have not been extensively studied. Although several studies have identified loss of heterozygosity in morphologically normal breast epithelium [45-47] and stroma [42,48] derived from breast cancer patients, other studies have proposed that these changes were distinct from the co-existing cancer [49]. Hence, it is unclear whether genomic alterations observed in morphologically normal breast tissues represent early precursors of breast cancer, markers of increased risk, or population based polymorphisms. In this paper, we present the most complete study to date of gene expression in normal breast tissues. Using LCM and whole genome microarray analysis we have characterized tissue-specific gene expression and identified markers of normal epithelium and stroma.

A primary goal of our study was to establish if a cancer-associated expression signature could be detected in morphologically normal breast tissues obtained from patients with breast cancer. Several approaches were used to address this question. First, we compared gene expression in morphologically normal tissue derived from breast cancer patients to that of healthy individuals undergoing breast reduction surgery. Second, we investigated if the pattern of gene expression in normal breast tissues derived from breast cancer patients was associated with clinical or pathological features of the corresponding cancer. A combination of class discovery, class distinction and class prediction approaches was used to analyze gene expression in microdissected epithelial and stroma samples (Figure 1). The results of this analysis demonstrate that microdissected samples clustered according to tissue type, and not according to the clinical or individual characteristics of the patients (Figures 2, 3 and 6). Moreover, our inability to identify statistically or biologically relevant predictors of the adjacent and reduction classes (Additional files 2 and 3) demonstrates that cancer-adjacent and breast reduction normal tissues have essentially homogeneous expression profiles. Furthermore, variations in gene expression between groups of samples are not associated with clinical characteristics but can be explained by tissue- and patient-specific variability. These data are in agreement with a previous study [12] that demonstrated a lack of significant differences between breast reduction and cancer-adjacent epithelium (three samples) using cDNA microarrays. In addition, our study now demonstrates a lack of significant differences between breast reduction and cancer adjacent stroma.

Notably, ER status, which is often the most important classifier of tumors, both clinically and at the molecular level [4,10], did not associate with any clusters observed in normal stroma or epithelium, nor were we able to identify any predictors for this clinical category. Identical approaches of class distinction, class prediction, and class discovery failed to identify biologically relevant or statistically significant predictors, or clusters associated with any of the other clinical characteristics tested (Additional files 2 and 3). These results suggest that, at the level of global gene expression, there is no significant cancer-associated expression signature detectable in normal breast tissues. We cannot, however, completely rule out the possibility that some subtle changes are present but are obscured by other effects, such as patient variability, or technical limitations.

While we were unable to identify predictors of clinical characteristics, there were genes differentially expressed between some of these clinical characteristics. In most cases the functional categories that were overrepresented consisted mostly of metabolic pathways and processes. Class discovery in normal adjacent stroma revealed two statistically significant clusters associated with stromal cellularity. While we were unable to identify a predictor of stromal cellularity, the differentially expressed genes identified in the class distinction were overrepresented in a number of interesting functional categories, including branching morphogenesis, endocytosis, neurogenesis, and patterning of blood vessels. For example, NOTCH4, a receptor for the Notch pathway that has been shown to inhibit angiogenesis [50], was elevated in the pauci cellular fibrotic stroma cluster when compared to the higher cellularity stroma, while JAG1, a Notch ligand shown to induce angiogenesis in some head and neck tumors [51], was elevated in highly cellular stroma compared to pauci cellular fibrotic stroma. Since we have been careful to sample stroma from the extralobular compartment, it is unlikely that these differences represent extralobular and intralobular stroma. However, we cannot rule out that these may be differences between stromal compartments that have previously not been identified based on morphology.

Comparison of our data to published data sets reveals the similarity of normal stroma and epithelium expression signatures with previously published gene expression profiles of epithelium and collective fibroblasts, endothelium, and myofibroblasts isolated from reduction mammoplasty samples [33]. Previous studies have examined the gene expression of cultured fibroblasts in response to serum and demonstrated that this expression program resembled that of a wound response [44] as well as expression profiles from tumors with fibroblastic features [36]. The serum/wound response expression profile was predictive of metastasis and progression in several carcinomas. Our normal breast stroma profile exhibits an expression pattern similar to unstimulated fibroblasts [44,52] and demonstrates that DTF tumors are more related to normal stroma than a SFT signature [36]. Since a DTF tumor profile has been shown to be associated with favorable outcome in breast tumors [36], the enrichment for DTF genes in our normal stroma profile is consistent with this finding.

Notably, clustering of a large breast cancer dataset [8] with the normal stroma and epithelium profile identified two significant clusters of samples (Figure 8). The smaller of the two clusters consisted of 38 samples, which were all identified as ER negative, HER2 negative, and PR negative. This cluster expressed genes specific to basal-like and normal-like cancer subtypes, including keratin-5, keratin-17, and GGH. The remaining ER negative samples were contained within the larger cluster of 266 samples. This cluster was composed of ER negative/HER2 positive, and ER positive/HER2 negative samples, which are characteristic of HER2 positive and luminal cancer subtypes, respectively [11,35]. Clustering of the cancer data using only epithelium specific genes led to repeated observation of a distinct basal-like cluster, whereas clustering using only stroma-specific genes led to co-clustering of the basal-like, ER positive, and HER2 positive tumors. This is in contrast to a recent report showing successful prognostic prediction in breast tumor microarray data using, amongst others, a stroma based signature [53]. The stroma based predictor used in that study was the wound response signature (similar to the CSR response signature), which we have shown is not expressed in normal stroma. Consequently, the predictive genes of the CSR (and wounding) signature are not selected as part of the intrinsic normal stroma signature, and thus we do not see association with prognosis when clustering using the intrinsic normal stroma genes.

**Figure 10:**
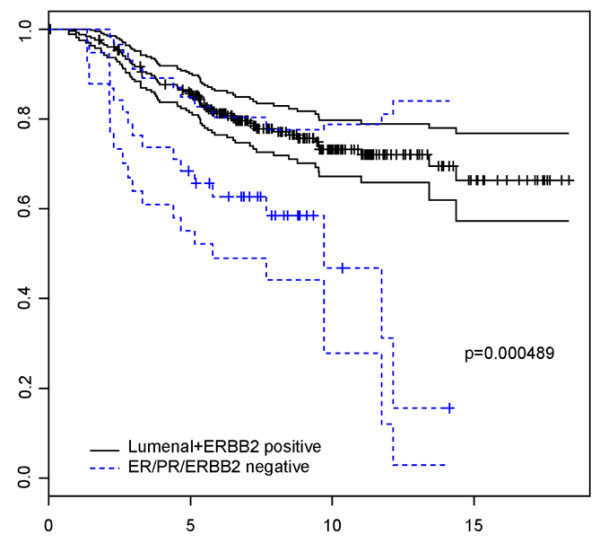
Survival analysis of the two sample clusters identified from the cancer data set [8]. The clusters were generated from the normal breast tissue signature. The estrogen receptor (ER)/progesterone receptor (PR)/ERBB2 negative cluster consisting of 38 samples shows poor survival compared to the remaining samples consisting of Lumenal and ERBB2 positive tumors (p = 0.000489).

A similar basal-like and normal-like cluster was identified using the intrinsic cancer gene set of Sorlie and colleagues [35]. This indicates that the basal-like and normal-like breast cancer subtypes are more similar to normal epithelial tissue than the other breast cancer subtypes. This is not entirely surprising, since normal ductal epithelium does not express high levels of ER, PR or HER2 [54,55]. When analyzed in a different cancer dataset, the basal-like subtype had a poor outcome when compared to other subtypes of breast cancer [35]. We also observed a poor outcome for the cluster of 38 ER/PR/HER2-negative samples compared to the larger cluster of ER positive, and HER2 positive samples (p = 0.000489, Figure 10). We found that this difference in survival could be explained primarily by the ER status of the sample (data not shown). The similarity of the basal-like and normal-like breast cancer subtypes has previously been shown by gene expression studies [10,11,35]. We have found that these subtypes are distinguished from ER positive and HER2 positive subtypes, at least in part, by the expression of epithelium-specific genes. In contrast, the HER2 positive and luminal subtypes exhibit enriched expression of stroma-specific genes. However, elevated expression of stroma-specific genes is not ubiquitous across all luminal or HER2 positive samples, nor is it correlated with any identifiable tumor subtypes (Figures 8 and 9). Nonetheless, these differences in stromal and epithelial expression drive the clustering of breast cancer subtypes using our normal breast tissue expression signature.

## Conclusion

This study provides the first in depth analysis of gene expression in morphologically normal epithelium and stroma adjacent to breast cancers as well as from reduction mammoplasty specimens. Analysis of the gene expression profiles revealed that there are no significant differences between tumor derived and reduction mammoplasty derived tissue. The analysis of these expression profiles in other breast cancer datasets identifies a distinct HER2/ER/PR negative subcluster that corresponds to a mixture of basal-like and normal-like cancer subtypes and reveals molecular similarities between normal breast epithelium and basal-like breast tumors with poor outcome. Moreover, the lack of any cancer-associated patterns of gene expression in morphologically normal breast tissues will enhance our understanding of early changes involved in cancer initiation. Furthermore, these data provide a base for the interpretation of breast cancer molecular profiling experiments and for the discovery of novel prognostic markers.

## Abbreviations

CSR = core serum response; DTF = desmoid type fibromatosis; ER = estrogen receptor; GGH = gamma-glutamyl hydrolase; GITC = guanidinium isothiocyanate; GO = Gene Ontology; LCM = laser capture microdissection; LIMMA = linear models for microarray analysis; PAM = prediction around medoids; PR = progesterone receptor; SAGE = serial analysis of gene expression SAM = significance analysis of microarrays; SFT = solitary fibrous tumor; TBS-T = tris-buffered saline tween-20.

## Competing interests

The authors declare that they have no competing interests.

## Authors' contributions

GF, SS, MP and MH wrote the paper. GF performed computational experiments and analyzed the microarray data. GF and FP performed quality control. SS established protocols for amplification and labeling of RNA isolated by LCM and supervised LCM, RNA isolation and labeling and microarray hybridizations. MS performed the immunohistochemistry experiments and collected the stroma cellularity data. FH, AO, and KK classified tissues for microdissection and reviewed the tissue pathology. BZ collected patient history. SM was instrumental in collecting tissue samples.

## Supplementary Material

Additional file 4 A table listing complete clinical characteristics of patients in this study. Click here for file

Additional file 8 A complete list of tissue specific expression markers identified in this study. Click here for file

Additional file 9 A complete list of GO categories overrepresented by the normal epithelium and normal stroma gene signatures. Click here for file

Additional file 3 A table listing tissue specific predictors of clinical characteristics based upon gene expression in adjacent epithelium. The poor quality of the predictors is readily visible from the error rate for the predictors in the first column of the table. The error rate is the fraction of times the predictor misclassifies a sample under cross-validation. Predictors were trained using gene sets from class distinction using SAM or LIMMA. For some combinations of clinical characteristics and class distinction algorithm, no genes passed the filtering criteria, and no predictor could be trained. In such cases the rows are omitted from the table. The gene set size is the initial size of the candidate gene set from which a predictor is built. This set is also selected under cross-validation. The training error is the rate of misclassification for samples included in the training set. The PAM cross-validation error rate reported by the PAM algorithm [30] does not account for the selection of the candidate gene set under cross-validation. The predictor size is the number of genes in the predictor. Click here for file

Additional file 10 A list of genes differentially expressed between cellular and pauci cellular fibrotic stroma clusters. Click here for file

Additional file 11 A list of GO terms overrepresented by genes differentially expressed between cellular and pauci cellular fibrotic stroma clusters. Click here for file

Additional file 2 A table listing tissue specific predictors of clinical characteristics based upon gene expression in adjacent stroma. The poor quality of the predictors is readily visible from the error rate for the predictors in the first column of the table. The error rate is the fraction of times the predictor misclassifies a sample under cross-validation. Predictors were trained using gene sets from class distinction using SAM or LIMMA. For some combinations of clinical characteristics and class distinction algorithm, no genes passed the filtering criteria, and no predictor could be trained. In such cases the rows are omitted from the table. The gene set size is the initial size of the candidate gene set from which a predictor is built. This set is also selected under cross-validation. The training error is the rate of misclassification for samples included in the training set. The PAM cross-validation error rate reported by the PAM algorithm [30] does not account for the selection of the candidate gene set under cross-validation. The predictor size is the number of genes in the predictor. Click here for file

Additional file 12 A figure showing principal component analysis of matched adjacent normal tissues. **(a) **Scree plot showing the percent of data variation explained by the first 10 principal components of the patient matched adjacent normal tissue. The common reference design accounts for 84.58% of variations in gene expression observed in the data (Additional file 13), while principal components 2 and 3 are explained by variations in gene expression associated with tissue type, and components 4 through 8 are explained by variations in gene expression between individuals. **(b) **Scatter plot of principal component two against principal component 3. These two dimensions suffice to summarize the between tissue variation observed in the data, as demonstrated by the clustering of epithelial samples on the right of the plot (red), and stromal samples on the left (black). Analogously, in five dimensions, we can explain the variation between individuals. No other clinical characteristics were significantly associated with any principal components. Click here for file

Additional file 13 A figure showing the effect of the common reference design in principal component analysis. Data that exhibit no variation in gene expression corresponds to an expression matrix where each gene on each array has exactly the same expression level. A slightly more realistic case exists where each gene has a different expression level, but the expression is just random noise (left panel). The principal components each explain a similar, small amount of the total variation in the data. The case at the other extreme of the spectrum from the random noise example consists of perfectly correlated data with no noise, as might be imagined from ideal replicate arrays (middle panel). The variability in the data occurs from each gene having a different level of expression; however, that expression is identical across arrays. Only one principal component is necessary to capture all of the variation in the data. The third and most realistic case consists of correlated data with random noise. This closely resembles what is observed in the normal tissue dataset with a common reference design. The arrays are highly correlated, resulting in the first principal component explaining the majority of the observed variations, and the remaining variation distributed amongst the remaining components. Click here for file

Additional file 6 A figure showing heatmaps of normal tissue expression profiles clustered using published gene signatures. **(a) **SFT signature, **(b) **DTF signature [36], **(c) **activated CSR signature, **(d) **inactive CSR signature [44]. Click here for file

Additional file 7 A schematic outlining the gene set comparisons and filtering operations performed using the normal tissue signature and gene sets from published expression profiles. Circles denote gene sets, labeled by name and with their size. Numbers in brackets denote the size of a gene set after filtering for high variance genes (Var >1) in normal tissue; 7.36% of genes in the normal dataset have variance greater than 1. Intersections between gene sets as well as the size of filtered gene sets are labeled with p values denoting the significance of the overlap (hypergeometric test), or the significance of overrepresentation of high variance genes (χ^2 ^goodness of fit test), respectively. The data were derived from the following sources: SFT/DTF (Additional file 6a,b) [36]; SAGE [33]; CSR (Additional file 6c,d) [44]. Click here for file

Additional file 1 A table listing p values for tests of association between clinical variables and top-level clusters (red boxes, Figure 6) induced by clustering various subsets of the data. Only normal adjacent stroma shows top-level clusters with significant p values by the bootstrap. None of the clinical variables were found to be correlated with either top-level clusters or statistically significant subclusters (data not shown). Click here for file

Additional file 5 A figure showing hematoxylin and eosin staining of **(a) **a breast reduction specimen and **(b) **a histologically normal specimen from an invasive breast carcinoma patient. Click here for file

## Figures and Tables

**Table 1:** Summary of clinical characteristics of patients sampled for this study

Characteristic	Number
Adjacent	34
Reduction	10
ER	
Positive	21
Negative	12
Normal	10
NA	1
Total	44
HER2	
Positive	8
Negative	22
Normal	10
NA	4
Total	44
PR	
Positive	13
Negative	20
Normal	10
NA	1
Total	44
Lymph node status	
Positive	14
Negative	20
Normal	10
NA	0
Total	44
Recurrence	
Positive	5
Negative	38
Normal	0
NA	1
Total	44
Menopausal status	
Post	16
Pre	13
Peri	1
NA	4
Surgical	10
Total	44
Age (mean ± SD)	52.18 ± 12.54
Tumor size (mean ± SD)	24.76 ± 14.06

ER, estrogen receptor; NA, not available; PR, progesterone receptor.

**Table 2:** Selected tissue markers identified for normal stroma and normal epithelium

Stromal expression	Epithelial expression	p value	Gene symbol	Gene name
24.41	1.00	1.02E-12	SFRP4	Secreted frizzled-related protein 4
18.44	1.00	1.02E-12	AOC3	Amine oxidase, copper containing 3 (vascular adhesion protein 1)
17.46	1.00	3.30E-11	PTGIS	Prostaglandin I2 synthase
17.11	1.00	7.97E-12	TEK	TEK tyrosine kinase, endothelial (venous malformations, multiple cutaneous and mucosal)
16.68	1.00	1.02E-12	IGFBP7	Insulin-like growth factor binding protein 7
15.65	1.00	1.02E-12	COL1A2	Collagen, type I, alpha 2
15.58	1.00	7.97E-12	WISP2	WNT1 inducible signaling pathway protein 2
14.37	1.00	1.94E-12	FBN1	Fibrillin 1
13.85	1.00	1.89E-11	CD36	CD36 antigen (collagen type I receptor, thrombospondin receptor)
1.00	2.37	8.32E-09	PPP1CB	Protein phosphatase 1, catalytic subunit, beta isoform
1.00	3.07	1.30E-08	K03200	Human melanoma-associated antigen p97
1.00	3.95	2.63E-09	PERP	TP53 apoptosis effector
1.00	4.08	7.50E-09	DDR1	Discoidin domain receptor family, member 1
1.00	4.75	2.11E-08	CDH1	Cadherin 1, type 1, E-cadherin (epithelial)
1.00	4.79	2.36E-07	KRT14	Keratin 14
1.00	5.14	1.60E-10	F11R	F11 receptor, junctional adhesion molecule 1
1.00	6.30	1.86E-07	KIT	v-kit Hardy-Zuckerman 4 feline sarcoma viral oncogene homolog
1.00	6.35	5.97E-08	KRTCAP3	Keratinocyte associated protein 3.
1.00	9.07	2.92E-08	ELF5	E74-like factor 5 (epithelium-specific Ets transcription factor 2)
