# Supplementary material for: Gene expression signatures of morphologically normal breast tissue identify basal-like tumors
Source: Breast Cancer Res. 2006 Oct 20;8(5):R58. doi: 10.1186/bcr1608 (PMC1779486; doi:10.1186/bcr1608)
Supplement: Additional file 4 — A table listing complete clinical characteristics of patients in this study. [file bcr1608-S4.pdf]

Patient	ER	Grade	HER2	PR	Lymph Node Status	Age	Tumor Size	Menopausal Status	Type	Recurrence	Cellularity
018	Positive	III	Positive	Negative	Positive	77	40	Post-menopause	Adjacent	Yes	Low
345	Positive	III	Positive	Negative	Negative	56	46	Post-menopause	Adjacent	No	-
136	Positive	III	Negative	Positive	Positive	48	90	Pre-menopause	Adjacent	No	-
190	Positive	III	Negative	Negative	Positive	63	30	Surgical menopause	Adjacent	No	-
295	Positive	III	Negative	Positive	Negative	46	24	Pre-menopause	Adjacent	No	-
301	Positive	III	Negative	Positive	Negative	60	18	-	Adjacent	No	Low
351	Positive	III	Negative	Negative	Negative	67	21	Post-menopause	Adjacent	No	Low
453	Positive	III	Negative	Positive	Negative	64	17	Post-menopause	Adjacent	No	Low
070	Positive	III	-	Positive	Positive	51	20	Surgical menopause	Adjacent	Yes	-
047	Positive	II	Positive	Positive	Positive	62	20	Post-menopause	Adjacent	No	High
057	Positive	II	Positive	Negative	Positive	54	20	Surgical menopause	Adjacent	No	-
076	Positive	II	Positive	Negative	Positive	60	35	Post-menopause	Adjacent	No	High
083	Positive	II	Positive	Negative	Negative	59	20	Post-menopause	Adjacent	No	-
063	Positive	II	Negative	Negative	Positive	54	22	Surgical menopause	Adjacent	No	-
223	Positive	II	Negative	Positive	Positive	49	12	Pre-menopause	Adjacent	No	-
281	Positive	II	Negative	Positive	Positive	53	35	Pre-menopause	Adjacent	No	-
409	Positive	II	Negative	Negative	Negative	64	18	Post-menopause	Adjacent	No	High
065	Positive	II	Negative	Positive	Negative	44	18	Surgical menopause	Adjacent	No	-
161	Positive	II	Negative	Negative	Positive	70	10	Surgical menopause	Adjacent	No	-
344	Positive	II	Negative	Positive	Negative	75	21	Surgical menopause	Adjacent	No	-
027	Positive	II	-	Positive	Negative	50	20	Post-menopause	Adjacent	No	-
366	Negative	III	Positive	Negative	Negative	46	17	Pre-menopause	Adjacent	No	Low
113	Negative	III	Positive	Negative	Positive	51	15	Surgical menopause	Adjacent	No	-
075	Negative	III	Negative	Negative	Negative	58	25	Post-menopause	Adjacent	Yes	-
086	Negative	III	Negative	Negative	Negative	43	20	Pre-menopause	Adjacent	Yes	-
259	Negative	III	Negative	Positive	Negative	32	20	Pre-menopause	Adjacent	No	Low
315	Negative	III	Negative	Negative	Positive	55	29	Peri-menopause	Adjacent	Yes	High
348	Negative	III	Negative	Negative	Positive	40	25	Pre-menopause	Adjacent	No	Low
359	Negative	III	Negative	Negative	Negative	56	19	Surgical menopause	Adjacent	-	Low
392	Negative	III	Negative	Negative	Negative	64	18	Post-menopause	Adjacent	No	-
122	Negative	III	Negative	Positive	Negative	60	26	Post-menopause	Adjacent	No	-
372	Negative	III	Negative	Negative	Negative	35	38	-	Adjacent	No	-
039	Negative	II	-	Negative	Negative	64	18	Post-menopause	Adjacent	No	-
247	-	II	-	-	Negative	67	15	Post-menopause	Adjacent	No	-
043	Normal	Normal	Normal	Normal	Normal	31	Normal	Pre-menopause	Reduction	No	High
132	Normal	Normal	Normal	Normal	Normal	27	Normal	Pre-menopause	Reduction	No	-
152	Normal	Normal	Normal	Normal	Normal	47	Normal	Pre-menopause	Reduction	No	-
153	Normal	Normal	Normal	Normal	Normal	27	Normal	-	Reduction	No	High
019	Normal	Normal	Normal	Normal	Normal	31	Normal	-	Reduction	No	High
036	Normal	Normal	Normal	Normal	Normal	48	Normal	Post-menopause	Reduction	No	-
044	Normal	Normal	Normal	Normal	Normal	44	Normal	Surgical menopause	Reduction	No	-
052	Normal	Normal	Normal	Normal	Normal	33	Normal	Pre-menopause	Reduction	No	-
193	Normal	Normal	Normal	Normal	Normal	57	Normal	Post-menopause	Reduction	No	-
101	Normal	Normal	Normal	Normal	Normal	54	Normal	Pre-menopause	Reduction	No	High

Supplementary Table S4:
