# Supplementary material for: Gene expression signatures of morphologically normal breast tissue identify basal-like tumors
Source: Breast Cancer Res. 2006 Oct 20;8(5):R58. doi: 10.1186/bcr1608 (PMC1779486; doi:10.1186/bcr1608)
Supplement: Additional file 2 — A table listing tissue specific predictors of clinical characteristics based upon gene expression in adjacent stroma. The poor quality of the predictors is readily visible from the error rate for the predictors in the first column of the table. The error rate is the fraction of times the predictor misclassifies a sample under cross-validation. Predictors were trained using gene sets from class distinction using SAM or LIMMA. For some combinations of clinical characteristics and class distinction algorithm, no genes passed the filtering criteria, and no predictor could be trained. In such cases the rows are omitted from the table. The gene set size is the initial size of the candidate gene set from which a predictor is built. This set is also selected under cross-validation. The training error is the rate of misclassification for samples included in the training set. The PAM cross-validation error rate reported by the PAM algorithm [30] does not account for the selection of the candidate gene set under cross-validation. The predictor size is the number of genes in the predictor. [file bcr1608-S2.pdf]

	Error Rate	Gene Set Size	Predictor Size	Training Error	PAM Cross-Validation Error
Menopausal Status - SAM	0.375 ± 0.25	83.25 ± 43.77	12.75 ± 4.19	0.16 ± 0.03	0.40 ± 0.04
Menopausal Status - Limma	0.5 ± 0	9.55 ± 3.14	6.91 ± 2.70	0.31 ± 0.04	0.47 ± 0.05
ER - Limma	0.5 ± 0.35	2651.78 ± 100.84	122 ± 105.15	0.038 ± 0.021	0.48 ± 0.08
PR - Limma	0.5 ± 0.24	910.4 ± 135.33	34 ± 27.68	0.079 ± 0.04	0.47 ± 0.17
HER2 - SAM	0.5 ± 0	65.33 ± 63.32	20.67 ± 8.08	0.13 ± 0.11	0.24 ± 0.05
HER2 - Limma	0.5 ± 0.32	54 ± 30.79	30.83 ± 13.56	0 ± 0	0.41 ± 0.16
Grade - Limma	0.4 ± 0.21	286.3 ± 49.52	78.4 ± 57.48	0.042 ± 0.028	0.39 ± 0.09
Adjacent vs Reduction - Limma	0.42 ± 0.20	7664.83 ± 27.82	28.5 ± 6.09	0 ± 0	0.18 ± 0.07

Supplementary Table S2:
