# Supplementary figures and images for: Gene expression signatures of morphologically normal breast tissue identify basal-like tumors
Source: Breast Cancer Res. 2006 Oct 20;8(5):R58. doi: 10.1186/bcr1608 (PMC1779486; doi:10.1186/bcr1608)

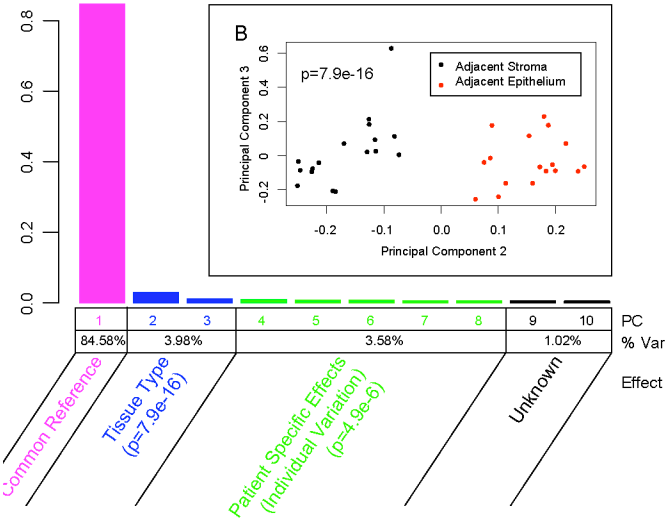

Figure S4

Supplement: Additional file 12 — A figure showing principal component analysis of matched adjacent normal tissues. (a) Scree plot showing the percent of data variation explained by the first 10 principal components of the patient matched adjacent normal tissue. The common reference design accounts for 84.58% of variations in gene expression observed in the data (Additional file 13), while principal components 2 and 3 are explained by variations in gene expression associated with tissue type, and components 4 through 8 are explained by variations in gene expression between individuals. (b) Scatter plot of principal component two against principal component 3. These two dimensions suffice to summarize the between tissue variation observed in the data, as demonstrated by the clustering of epithelial samples on the right of the plot (red), and stromal samples on the left (black). Analogously, in five dimensions, we can explain the variation between individuals. No other clinical characteristics were significantly associated with any principal components. [file bcr1608-S12.pdf]

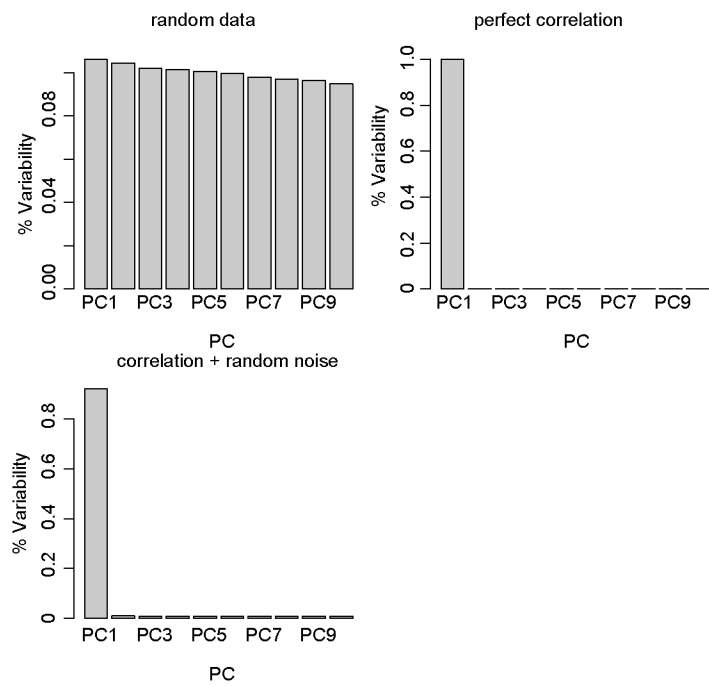

Figure S5

Supplement: Additional file 13 — A figure showing the effect of the common reference design in principal component analysis. Data that exhibit no variation in gene expression corresponds to an expression matrix where each gene on each array has exactly the same expression level. A slightly more realistic case exists where each gene has a different expression level, but the expression is just random noise (left panel). The principal components each explain a similar, small amount of the total variation in the data. The case at the other extreme of the spectrum from the random noise example consists of perfectly correlated data with no noise, as might be imagined from ideal replicate arrays (middle panel). The variability in the data occurs from each gene having a different level of expression; however, that expression is identical across arrays. Only one principal component is necessary to capture all of the variation in the data. The third and most realistic case consists of correlated data with random noise. This closely resembles what is observed in the normal tissue dataset with a common reference design. The arrays are highly correlated, resulting in the first principal component explaining the majority of the observed variations, and the remaining variation distributed amongst the remaining components. [file bcr1608-S13.pdf]

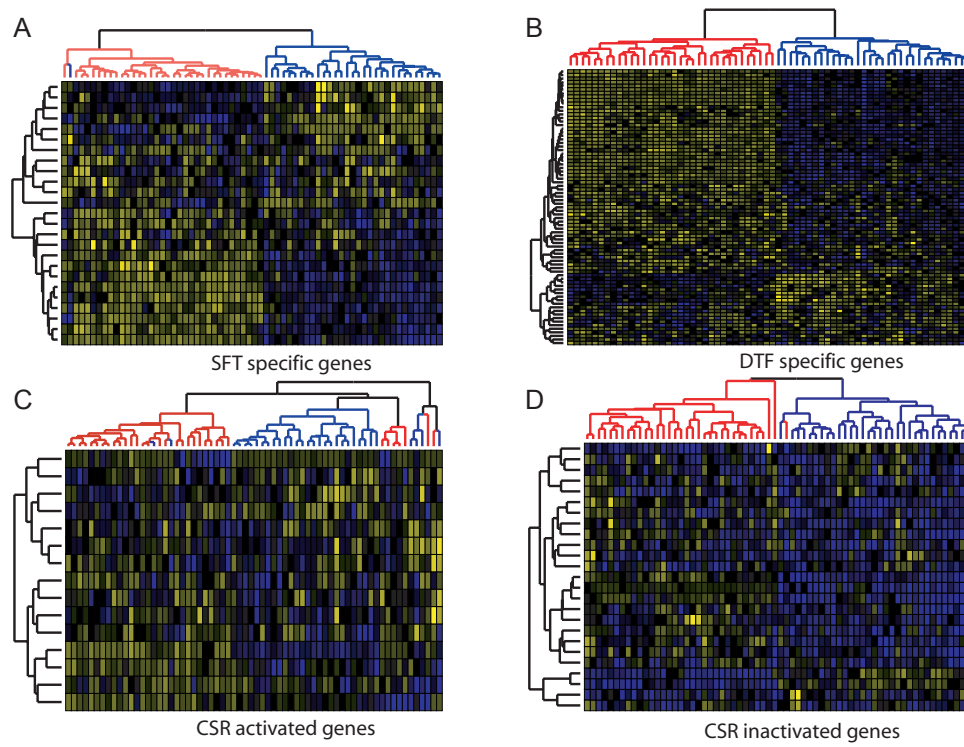

Figure S2

Supplement: Additional file 6 — A figure showing heatmaps of normal tissue expression profiles clustered using published gene signatures. (a) SFT signature, (b) DTF signature [36], (c) activated CSR signature, (d) inactive CSR signature [44]. [file bcr1608-S6.pdf]

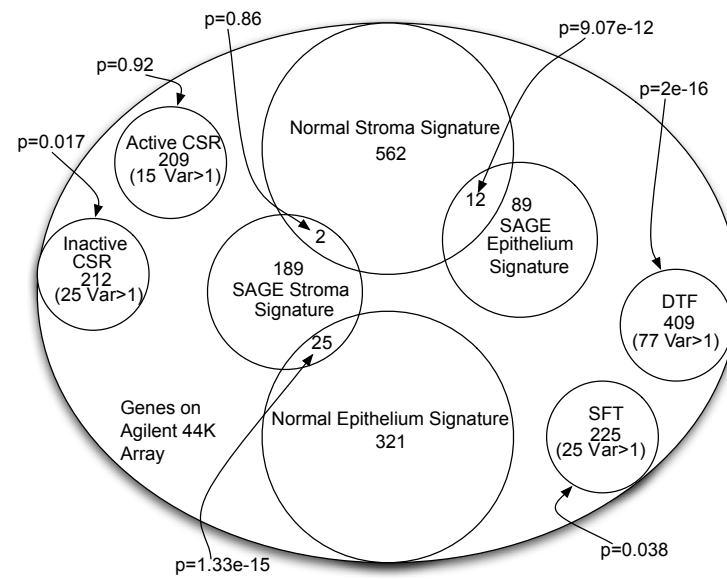

Figure S3

Supplement: Additional file 7 — A schematic outlining the gene set comparisons and filtering operations performed using the normal tissue signature and gene sets from published expression profiles. Circles denote gene sets, labeled by name and with their size. Numbers in brackets denote the size of a gene set after filtering for high variance genes (Var >1) in normal tissue; 7.36% of genes in the normal dataset have variance greater than 1. Intersections between gene sets as well as the size of filtered gene sets are labeled with p values denoting the significance of the overlap (hypergeometric test), or the significance of overrepresentation of high variance genes (χ2 goodness of fit test), respectively. The data were derived from the following sources: SFT/DTF (Additional file 6a,b) [36]; SAGE [33]; CSR (Additional file 6c,d) [44]. [file bcr1608-S7.pdf]

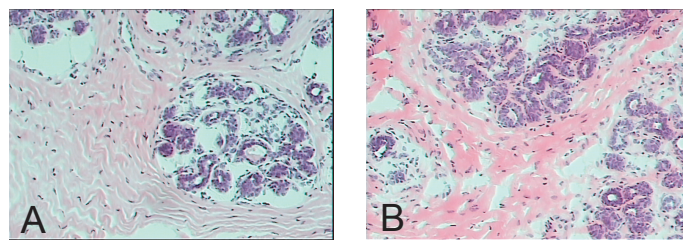

Figure S1

Supplement: Additional file 5 — A figure showing hematoxylin and eosin staining of (a) a breast reduction specimen and (b) a histologically normal specimen from an invasive breast carcinoma patient. [file bcr1608-S5.pdf]
