# Supplementary material for: Gene expression signatures of morphologically normal breast tissue identify basal-like tumors
Source: Breast Cancer Res. 2006 Oct 20;8(5):R58. doi: 10.1186/bcr1608 (PMC1779486; doi:10.1186/bcr1608)
Supplement: Additional file 1 — A table listing p values for tests of association between clinical variables and top-level clusters (red boxes, Figure 6) induced by clustering various subsets of the data. Only normal adjacent stroma shows top-level clusters with significant p values by the bootstrap. None of the clinical variables were found to be correlated with either top-level clusters or statistically significant subclusters (data not shown). [file bcr1608-S1.pdf]

Variable	Adjacent + Reduction Epithelium (Fig 5, panel A)	Adjacent Epithelium (Fig 5, panel B)	Adjacent + Reduction Stroma (Fig 5, panel C)	Adjacent Stroma (Fig 5, panel D)
Lymph Node Status	0.656	1.00	0.692	1.00
ER	0.393	0.673	1.00	1.00
Grade	1.00	0.671	0.689	0.226
HER2	1.00	0.613	0.643	1.00
PR	0.669	1.00	0.689	0.688
Age of Operation	0.704	0.297	0.356	0.479
Tumor Size	0.253	0.113	0.240	0.513
Menopausal Status	0.266	0.589	0.494	0.288
Recurrence	0.0526	0.116	1.00	0.645
Cellularity	NA	NA	0.0051	0.000209

Supplementary Table S1
